# Ergonomic human-robot collaboration in industry: A review

**DOI:** 10.3389/frobt.2022.813907

**Published:** 2023-01-03

**Authors:** Marta Lorenzini, Marta Lagomarsino, Luca Fortini, Soheil Gholami, Arash Ajoudani

**Affiliations:** ^1^ Human-Robot Interfaces and Physical Interaction Laboratory, Italian Institute of Technology, Genoa, Italy; ^2^ Neuroengineering and Medical Robotics Laboratory, Department of Electronics, Information and Bioengineering, Polytechnic University of Milan, Milan, Italy

**Keywords:** human-robot collaboration, ergonomics, human factors, human-robot interaction, collaborative robots, industry

## Abstract

In the current industrial context, the importance of assessing and improving workers’ health conditions is widely recognised. Both physical and psycho-social factors contribute to jeopardising the underlying comfort and well-being, boosting the occurrence of diseases and injuries, and affecting their quality of life. Human-robot interaction and collaboration frameworks stand out among the possible solutions to prevent and mitigate workplace risk factors. The increasingly advanced control strategies and planning schemes featured by collaborative robots have the potential to foster fruitful and efficient coordination during the execution of hybrid tasks, by meeting their human counterparts’ needs and limits. To this end, a thorough and comprehensive evaluation of an individual’s ergonomics, i.e. direct effect of workload on the human psycho-physical state, must be taken into account. In this review article, we provide an overview of the existing ergonomics assessment tools as well as the available monitoring technologies to drive and adapt a collaborative robot’s behaviour. Preliminary attempts of ergonomic human-robot collaboration frameworks are presented next, discussing state-of-the-art limitations and challenges. Future trends and promising themes are finally highlighted, aiming to promote safety, health, and equality in worldwide workplaces.

## 1 Introduction

It is common knowledge nowadays that workers, worldwide, are exposed to occupational risk factors that may have negative effects on their physical and mental health. Activities such as heavy material handling, repetitive movements and prolonged awkward sitting impose physical burden on workers’ bodies, resulting in the so-called musculoskeletal disorders (MSDs) (Pascual and Naqvi, 2008). Despite the extensive prevention efforts of the industrial world and the regulatory bureaus, these remain the most widespread work-related health problem in the European Union (EU). According to the European Agency for Safety and Health at Work (EU-OSHA), approximately three out of five workers suffer from an MSD, among which backache and upper limb pain are the most common (de Kok et al., 2019). Recent studies have also highlighted the importance of considering stress and psychosocial factors along with the aforementioned physical solicitations proposing a holistic approach. Indeed, results from the sixth European Working Condition Survey (EWCS) claim that 25% of European workers reported that their occupations have negative impacts on their mental and emotional state (Kubicek et al., 2019). Overall, besides the harmful effects on workers themselves, physical and mental health problems may lead to impressive costs to enterprises and society, being one of the most common causes of disability, sick leave and early retirement (Hassard et al., 2014; James et al., 2018).

In light of this, it becomes crucial for enterprises, trade unions and regulating authorities to address in an objective manner the hazardous factors that may lead to physical and mental distress among the workforce. Ergonomics studies and interventions can be classified into “microergonomics” and “macroergonomics.” The field of “macroergonomics” concentrates on designing overall work systems and determining how effective the technological and personnel sub-systems are with respect to external demands (Hendrick and Kleiner, 2002). In this review, we focus on the individual dimension of the workers, namely the analysis of worker postures, workplace productivity, work physiology and biomechanics within the scope of “microergonomics.” It is worth specifying that logistical and organisational aspects, such as resources allocation, shift/turn planning, and outline of the working environment, are not deeply examined in the text, but their impact on “microergonomics” is presented in the discussion.

**FIGURE 1:**
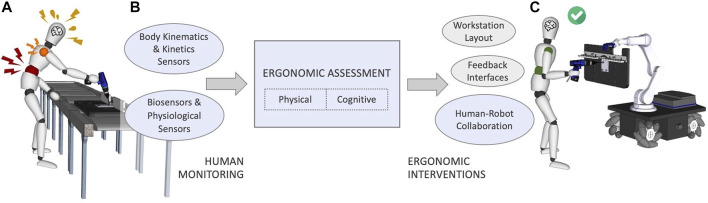
A conceptual illustration of a worker accomplishing an industrial task without **(A)** and with **(C)** a collaborative robotic solution. The structure of this article is also described in the schema **(B)**, highlighting (blue) the research themes that we focus on.

A thorough ergonomics assessment builds the foundation for a safer, healthier and less injury-prone workplace, resulting in an overall improvement of operators’ well-being. The key objective is to identify the risk factors and to quantify them, which can serve as a valuable tool to train the workforce. To enhance the ergonomic condition and awareness of the workers, researchers investigated several possible solutions that may be grouped in the following macro-areas: 1) effective design of comfortable and adjustable workstations (Shikdar and Hadhrami, 2007; del Rio Vilas et al., 2013; Peruzzini et al., 2019; Bongiovanni et al., 2022), 2) development of intuitive feedback interfaces that warn about risks and hazards (Villani et al., 2018a; Kim et al., 2018b, 2021a), and 3) creation of advanced human-robot shared workstations for fruitful and ergonomic collaboration in hybrid environments (Kim et al., 2018a; El Makrini et al., 2019). As conceptually illustrated in Figure 1, the latter is the key topic of this article.

This last scenario falls within the general term human-robot interaction (HRI) and is probably on the cutting edge among the industrial research topics. This article is focused on human-robot collaboration (HRC) as opposed to HRI since these two terms hold different meanings. Interaction determines any kind of action that involves another human being or robot, who does not necessarily profit from it. On the other hand, humans and robots collaborating on a shared task form a team. A team is defined as a small number of partners with complementary skills who are committed to a common purpose, performance goal, and approach. The same holds for human-robot teams where the partners are humans and robots, committed to reach a joint objective through collaboration. The advent of collaborative robots (CoBots) broadened the application possibilities attracting the attention of research community. CoBots can indeed support their human counterparts in performing physical (e.g., relieving the workers from part of the effort while handling heavy loads (Brosque et al., 2020)), cognitive (e.g., visualising alternative behaviours to reduce operators’ mental stress (Krupke et al., 2018)) and hazardous (e.g., handling chemical material (Liu and Wang, 2020)) operations. This relationship positively impacts productivity, flexibility, and the creation of new jobs instead of replacing workers. The previous referred studies demonstrated a link between CoBots and improved work conditions; however, the inclusion of ergonomic criteria in the development and implementation of these technologies is far from being well-known. In this article, the authors are interested in understanding studies that included ergonomics as a requirement in HRC systems, giving particular attention to the segmentation between physical and cognitive ergonomics, and presenting an offline or online application.

Multiple review articles on the integration of human factors and ergonomics in engineering and manufacturing processes design (Kolus et al., 2018; Sun et al., 2019) and, more in general, within Industry 4.0 (Kadir et al., 2019; Sgarbossa et al., 2020) have been proposed. Critical socio-technical factors for the successful implementation of Industry 4.0 were examined by Sony and Naik (2019, 2020). On the other hand, multiple review articles exist on HRC frameworks in industrial environments but focus primarily on technology development and determining and minimizing its intrinsic safety risks. An exhaustive review on HRC in industrial environment is provided by Villani et al. (2018a), with specific focus on issues related to physical (safety) and cognitive (intuitiveness-of-use) interaction. In fact, first, the safety standards are recollected to discuss the permitted interaction level between human and the robotic agents based on the introduced measures. Second, they inspect the user-interfaces, in the sense of cognitive workloads, claiming the traditional lead-through and offline programming are still the most used interfaces in industrial practice, despite the rise of more intuitive methods such as multi-modal interaction and extended reality technologies (e.g., virtual and augmented realities). They conclude the review by listing the commercially-available solutions, and their applications in the industrial setups to improve the efficiency of the conventional systems. Still, the roles and effects of HRC setups on the improvement of ergonomics, especially physical ergonomics, is not discussed in this article. Another interesting survey is presented in Kumar et al. (2020). The authors abstractly categorise the HRC setups into three main aspects, 1) awareness: level of perception using sensor information coming from human-operator, robots, and workspace, 2) intelligence: development of algorithms to achieve the robot’s actions and behaviours, and 3) compliance: dealing with the management of human expectation and communication between the agents. In particular, safety, trust-in-automation, and productivity factors are comprehensively discussed in the introduced intelligence category covering the latest research done in these regards. Nevertheless, here, the ergonomics aspects are not investigated. The available mechanisms to assure safety of CoBot systems in manufacturing is also discussed in Bi et al. (2021); Zacharaki et al. (2020). In addition, Castro et al. (2021) mostly focus on the current HRC research trends and their future directions. They claim that better interactions, cognitive integration, and the presence of effective metrics are the fundamental necessities of the future developments. The ergonomics research trends are focused in Gualtieri et al. (2021b); however, the corresponding ergonomics assessment and monitoring tools, and their effects on the HRC setups are not highlighted in this survey. Moreover, an interesting survey is presented in Berg and Lu (2020) that introduces the available user-interfaces for HRC but without studying the inherent ergonomics factors. In summary, What is missing is a review paper that tackle the inclusion and integration of human ergonomics principles specifically into human-robot collaborative solutions.

In an attempt to fill in the gap in the previously mentioned surveys, in this article, we mainly focus on the works that explicitly address human factors and ergonomics within HRC solutions. Among all the HRC possibilities, we specifically consider those frameworks in which a CoBot interacts with the human to accomplish a shared task, by adapting online^1^ its behaviour to address the counterpart’s demands. As such, teleoperation systems and exoskeletons are not covered here. The foremost objective of this article is to provide a *narrative review* of the current state-of-the-art in HRC to improve online human ergonomics in the industrial sector, and to highlight the most important and promising research themes identified for both physical and cognitive ergonomics.

The process we implemented to carry out the review is the following. We conducted an automatic search for papers that contain the selected keywords (which will be specified for each subtopic/Section) in Google Scholar and Scopus, since they are the most well-known and used Database in the target audience. Existing ergonomics assessment tools were investigated starting from the earlier reports in the field. Instead, the papers on ergonomics HRC were selected from 2011 until the moment of paper preparation (August 2022). We then meticulously examined the list of potentially relevant papers and excluded those that did not explicitly use/study the topic of interest or those that only mentioned it in the literature review part of the introduction. When multiple papers presented the same/similar idea, we selected the one published first, or in the case of evolved idea, we chose the journal version. The exceptions were some preliminary conference publications of the work that was later evolved and published in a journal for the purpose of historic narrative. In addition, we used several more general ergonomics and HRC papers to establish a context, introduce basic concepts, and support our statements.

The rest of the paper is organised as follows. Section 2 provides an overview of the of the existing ergonomics assessment tools to evaluate both physical and cognitive workload. The available technologies to monitor the human state are presented in Section 3, which may be beneficial to automatise ergonomics evaluation. In Section 4, preliminary attempts of ergonomic HRC frameworks are illustrated and in Section 5, state-of-the-art limitations and challenges are discussed. Last, the future trends are highlighted and the conclusions of the article are drawn in Section 6.

## 2 Ergonomics assessment in industrial settings

Due to the alarming statistics on workers’ health conditions in the industrial sector, various methods and approaches were developed, in the last decades, to assess the exposure to risks in the workplace. Chemical and environmental agents are not covered here, but we tackle those short or long-term factors that induce a hazardous workload on the operators. This section provides an overview of the studies wherein tools to evaluate ergonomics and human factors were developed and presented, categorising them according to the class of risk factors they seek to address. Specifically, the approaches that tackle physical workload are illustrated in Section 2.1 while studies on the psychosocial/organisational determinants, defined as “cognitive” workload, are outlined in Section 2.2. To focus the literature analysis on the assessment of workers’ ergonomics in industrial settings, we combined terms associated with workload (e.g., “physical,” “cognitive,” “workload,” “stress,” “effort,” “workplace”) with terms related to its analysis (e.g., “human factors,” “ergonomics,” “assessment,” “evaluation”) and we discard the studies dedicated to office work or services sectors. Indeed, most of the literature concentrates on factory jobs. Earlier reports (Burdorf, 1992; van der Beek and Frings-Dresen, 1998) on the assessment of physical workload conventionally categorised the methods in the following three groups:

• *subjective judgements*: self-questionnaires from workers or narrative interviews from experts;
• *systematic observations*: collected on-site at the workplace or from video recordings;
• *direct measurements*: performed on-site at the workplace or during simulations in laboratories.

**FIGURE 2:**
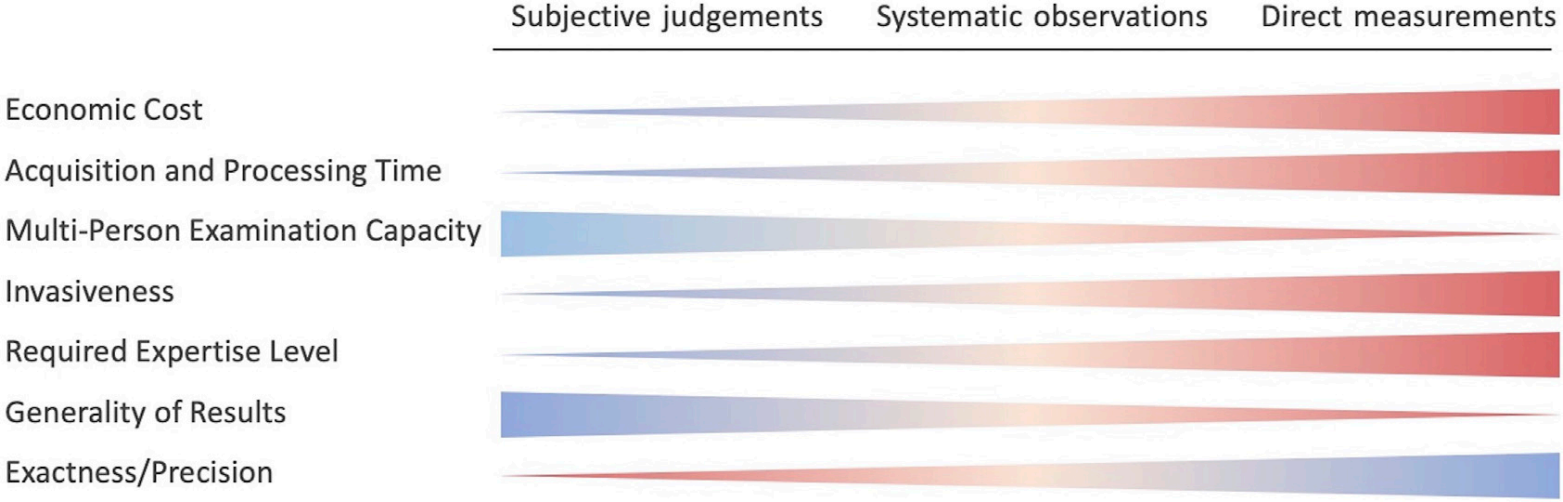
Overview of trade-offs among the different categories of methods for the assessment of physical and cognitive workload. The variation in thickness indicates the feature trend, while the colours reflect the positive (blue) or negative (red) impact.

Subjective measurements are surveys that can be completed either by the tested subjects or by an interviewer but always reflect the point of view of the former. With *systematic observations*, we refer to those procedures (e.g., worksheet to be filled in, parameters to be collected) that are carried out by experts and are based on simple observations of the examined subjects. In recent times, many of the methods belonging to this category have been automatised by leveraging the benefits of sensor technologies, but their use is not strictly necessary. Conversely, the techniques pertaining to *direct measurements* inevitably imply a sensor system due to the required accuracy and online availability of the measurements. The ergonomics assessment can then be extracted directly from the collected data or estimated by integrating them within ad-hoc models. The same categorisation may be applied and are adopted here for cognitive load measurements. The trend of some peculiar features, varying among these three groups in an orderly way, is represented in Figure 2.

### 2.1 Physical workload

In the earliest studies on the assessment of physical workload (Winkel and Mathiassen, 1994; Westgaard and Winkel, 1996), the authors introduced the term “mechanical exposure” to denote all the factors connected to the biomechanical forces generated into the human body when performing a work task. In this review, we embrace the same concept, thus not considering the full physical working environment (lighting, noise, thermal environment, etc.). Adopting the general model proposed by van der Beek and Frings-Dresen (1998), which describes how the working situation induces responses and health effects in the workers, we can distinguish two types of physical exposure: external and internal exposure. External exposure refers to the work environment and the actual working method, i.e. adopted postures, executed movements, and exerted forces that workers exploit to perform an activity with their anthropometric characteristics. The corresponding moments and forces within the human body are instead the internal exposure.

**FIGURE 3:**
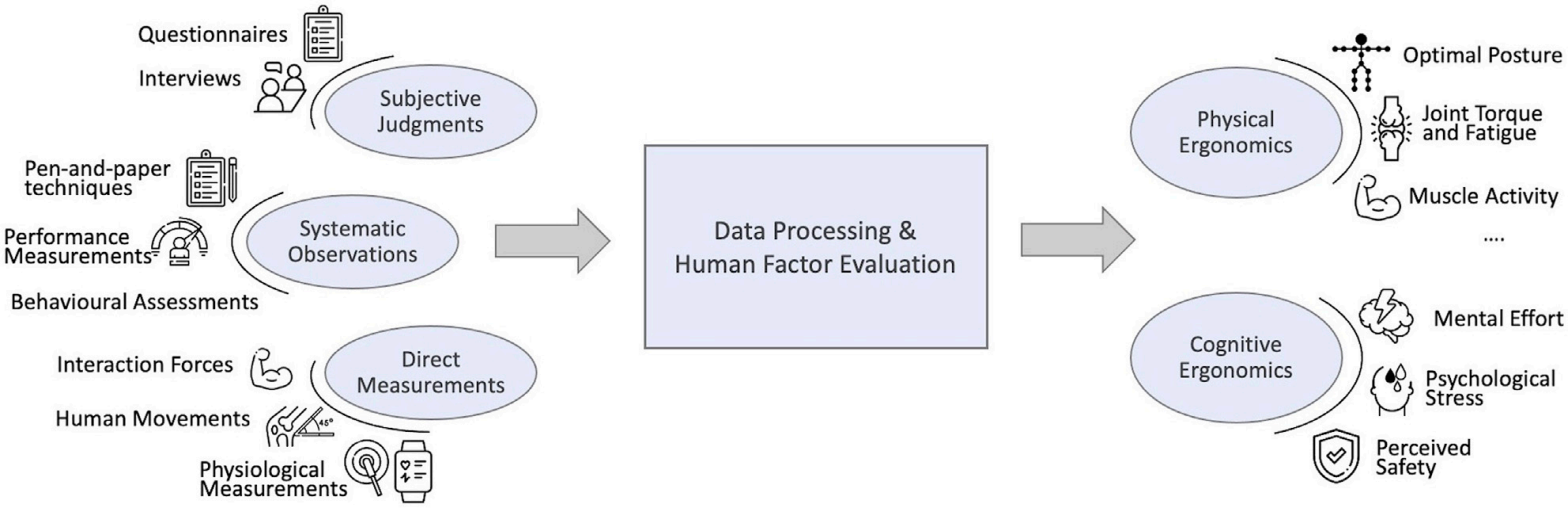
Overview of the ergonomic assessment methods to address both physical and cognitive workload, applicable to industrial settings.

Among the groups defined above and illustrated in Figure 3, *subjective judgements* and *systematic observations* are determined to tackle external exposure. The vast majority of tools employed in the current industrial scenario to assess workers’ ergonomics relies on such two categories, which are covered in Sections 2.1.1, 2.1.2. The methods that they gather are presented building on some comprehensive reviews (Li and Buckle, 1999; David, 2005; Marras, 2006; Andreas and Johanssons, 2018), in which ergonomics tools were listed, classified, and compared. On the other hand, *direct measurements* can be employed to estimate internal exposure. The corresponding category is addressed in Section 2.1.3.

#### 2.1.1 *Subjective judgements*

To assess physical workload, body discomfort, or job stress, it is possible to directly query the workers, investigating both physical and psychosocial factors. These methods take the form of body map (Corlett and Bishop, 1976), rating scales (Shackel et al., 1969; Borg et al., 1985), checklists (Drury and Coury, 1982; Cox and Mackay, 1985; Keyserling et al., 1992), and questionnaires or interviews (Kuorinka et al., 1987; Bigos et al., 1991; Dickinson et al., 1992; Wiktorin et al., 1993), among which the National Aeronautics and Space Administration—task load index (NASA–TLX) (Hart and Staveland, 1988; Hart, 2006) is the most used. Almost all the strategies for *subjective judgements* developed up to now were built on the basis of the above-mentioned earliest attempts. Traditionally, data were collected using written means, but more recent innovations include web-based facilities. The methods based on *subjective judgements* have the benefit of being straightforward to use (no specific expertise is required), applicable to a broad range of work situations and appropriate for surveying high numbers of subjects at comparatively low cost. Nevertheless, they are vulnerable to many influences and several studies have shown that they have too low validity (Burdorf and Laan, 1991) and reliability (Wiktorin et al., 1993) with respect to the demands for ergonomic interventions.

#### 2.1.2 *Systematic observations*

Several approaches were developed in the last decade to systematically record workplace exposure to be examined by an observer and stored on ad-hoc sheets. These are commonly referred to as “pen-and-paper”^2^ methods. Most of them have been conceived on the basis of the two most relevant and widespread normative aiming to establish ergonomic recommendations for workers, i.e., the International Organization for Standardization (ISO) 11228 and the European Standards (EN) 1005. The postures and movements of the workers can be carefully evaluated by a number of indicators: Posturegram (Priel, 1974), Posture targeting (Corlett et al., 1979), ovako working posture analysing system (OWAS) (Karhu et al., 1977), rapid upper limb assessment (RULA) (McAtamney and Corlett, 1993), hand-arm-movement analysis (HAMA) (Christmansson, 1994), a method assigned for the identification of ergonomics hazards (PLIBEL) (Kemmlert, 1995), quick exposure check (QEC) (Li and Buckle, 1998), and rapid entire body assessment (REBA) (Hignett and McAtamney, 2000). These techniques are relatively inexpensive to carry out and can be used in different work situations without hindering the workers, but they are applicable only to rather static or repetitive jobs.

Besides postures, other workplace factors such as load/force, repetition, duration of movement, vibration, and their interaction/combination have to be considered. Similar to these techniques for posture analysis, muscle fatigue analysis (MFA) (Rodgers, 2004) was proposed, whereby each body part is scaled into four effort levels according to its working position, but also to the duration of the effort and the frequency. Considering lifting and carrying loads, National Institute for Occupational Safety and Health (NIOSH) equations (Waters et al., 1993) were introduced to define the suggested load weight limit to be lifted by human operators considering gender, forces exerted on the spine structure, and calories consumed during the effort. The Washington Industrial Safety and Health Act (WISHA) lifting calculator was then developed based on NIOSH. Mechanical exposure can then be evaluated with respect to intensity (or magnitude), repetitiveness and duration even with the strain index (SI) (Steven Moore and Garg, 1995) and its revised versions (Garg et al., 2017), the American Conference of Governmental Industrial Hygienists—threshold limit values (ACGIH–TLV) (ACGIH, 1981), an assessment technique for postural loading on the upper body based on joint motion discomfort and maximum holding time (LUBA) (Kee and Karwowski, 2001), hand arm risk assessment method (HARM) (Douwes and Kraker, 2009) and manual handling assessment charts (MAC) (Monnington et al., 2003). Snook and Ciriello (1991) proposed a detailed procedure to assess the exerted force to perform pushing and pulling activities, taking into account the weight/distance of the handled object, the frequency, and duration of the action. Finally, some methods were focused on actions performed at high frequency with low loads and consider even the recovery time like occupational repetitive actions (OCRA), a concise index for the assessment of exposure to repetitive movements of the upper limb (Occhipinti, 1998). Although these indices are more exhaustive and have been widely adopted both by practitioners and researchers, they lack of precision and reliability and subjective variability can influence their results. Moreover, they do not provide a consistent and overall measure of the ergonomic risk since every index focuses on a specific manual material activity. In view of this, the ergonomic assessment worksheet (EAWS) method (Schaub et al., 2013) was developed to provide a unique and comprehensive ergonomic analysis. The EAWS comprises different sections (postures and movements, action forces, manual material handling, and upper limb, respectively) whose outcome can be integrated into a final score. Similarly, with the composite ergonomics risk assessment (CERA) (Szabò and Dobò, 2018) technique, a unified evaluation can be obtained after a separate determination of the different ergonomic risks, also based on workplace history. Lastly, the key indicator method (KIM) was introduced to tackle manual handling operations (KIM-MHO) (Klussmann et al., 2017), lifting, holding and carrying (KIM-LHC) and pulling and pushing (KIM-PP) (Steinberg, 2012).

Nevertheless, all these pen-and-paper techniques must be employed by trained experts as an offline procedure after collecting observations/recordings, which is rather time-consuming and does not provide immediate results. For this reason, several attempts were made to automatise the completion of some of the worksheets mentioned above to perform an online ergonomics evaluation. Specifically, the RULA (Shaikh et al., 2003; Ray and Teizer, 2012; Vignais et al., 2013; Haggag et al., 2013; Puthenveetil et al., 2015; Plantard et al., 2017), the REBA (Busch et al., 2017; Van de Perre et al., 2018) and the EAWS (Bortolini et al., 2018; Malaise et al., 2019) were considered, respectively, and combined with human motion data in both virtual and real environments. However, the main limitation of the observational methods still stand, i.e., the dynamics of the tasks are considered to a limited extent (e.g., interaction forces are considered constant).

#### 2.1.3 *Direct measurements*

To address humans’ physical internal exposure, *direct measurements* collected on the human subjects through suitable sensor systems were generally integrated with more or fewer complex models of the human body. Several algorithms were proposed for estimating muscle tensions and joint loads using detailed models of the human musculoskeletal system. One of the most well-known is the algorithm underlying the open-source software “OpenSim” (Delp et al., 2007). This platform enables the creation of dynamic simulations of movement that integrates off-the-shelf models describing the anatomy and physiology of the elements of the neuromusculoskeletal system and the mechanics of multi-joint movement. Similar capabilities are offered by the simulation software “Anybody” (Damsgaard et al., 2006), which is capable of analysing the musculoskeletal architecture of humans as rigid-body systems. Hence, standard methods of multi-body dynamics (i.e., inverse kinematics and inverse dynamics) can be applied but integrating into the model a reasonable representation of the muscle geometry and the recruitment pattern of the muscles. An analogous package is virtual interactive musculoskeletal system (VIMS) (Chao et al., 2007). Besides the massive studies behind the development of these platforms, there are also some minor works whereby muscle models were introduced to account for internal exposure (Bhargava et al., 2004; Forster, 2004; Nakamura et al., 2005; Fraysse et al., 2009; Millard et al., 2013). All the above-mentioned platforms and methodologies account for joint reactions (forces and torques) from motion data by using inverse dynamics and then optimisation techniques to compute the muscle tensions. Nevertheless, due to the actuation redundancy (the number of muscles is greater than the number of degrees of freedom (DoFs) of the system), a desired motion in terms of joint torques can be achieved by an infinite number of activation patterns of the muscles. Another drawback is that the complex musculoskeletal models underlying require the identification of numerous parameters (Ayusawa et al., 2014). Alternatively, they can be obtained by means of anthropometric standards and tables (Herman, 2007; Winter, 2009) thus they are not subject-specific.

An alternative solution is to measure muscle activation using electromyography (EMG) directly and exploit empirical models (Hill, 1938; Stroeve, 1999) to convert such activation into muscle tensions (Buchanan et al., 2004). One of the first attempts at using an EMG-based technique for the monitoring of low back physical exposure and cumulative compression was made by Mientjes et al. (1999). Afterwards, experimentally recorded EMG signals have been used in several studies to directly drive simulations of upper (Manal et al., 2002; Village et al., 2005; Nikooyan et al., 2012; Pau et al., 2012) and lower (Lloyd and Besier, 2003; Kumar et al., 2012; Manal et al., 2012; Sartori et al., 2012; Meyer et al., 2017) extremity musculoskeletal models. Whole-body muscle tension estimation by means of optical motion-capture and EMG measurements was enriched with a visual feedback interface in (Murai et al., 2010). Nevertheless, even EMG-based approaches incorporate numerous parameters and the use of EMG presents several drawbacks. The correct placement of EMG sensors is quite difficult and their relative movement in dynamic conditions makes the estimates questionable (Farina and Merletti, 2001). EMG signals are inevitably affected by various noise signals or artifacts (De Luca et al., 2010). Finally, many EMG-based techniques are conceived for specific body parts. As such, due to the inner complexity and reduced practicability, the methods based on *direct measurements* have been implemented nearly entire in laboratory settings.

Aiming to meet contemporary industry demands, some innovative approaches have been recently proposed. The latter evaluate human physical workload by relying on the online monitoring of human kinodynamic^3^ state through reduced-complexity estimation algorithms. The objective is to also account for the workers’ internal exposure, which is neglected by traditional ergonomic tools, while overcoming the limitation of the laboratory-based methods, impractical and hardly customisable. For instance, Maurice et al. (2017) proposed multiple ergonomic indicators that are capable of quantifying exhaustively and concisely the physical demands endured by a worker when executing various manual activities, addressing both postures/movements and forces/torques. By exploiting the principles of humanoid robotics to model human kinodynamics, Lorenzini et al. (2022) proposed an online multi-index approach to account for multiple potential contributors to MSDs, also giving importance to the subject-specific requirements of the workers. In the same line, Gholami et al. (2022) introduced a set of quantitative metrics to take into account operators’ ergonomics in the assessment of teleoperation interfaces. Still chasing a reduced-complexity approach but adding a certain level of accuracy for more sophisticated body districts, Ventura et al. (2021) developed a flexible model of the human spine mechanics for assessing compressive loading. Finally, Latella et al. (2019) presented a stochastic methodology for the simultaneous floating-base estimation of the human whole-body kinematics and dynamics toward online ergonomics assessment. Nevertheless, this approach is thus far rather limited.

### 2.2 Cognitive workload

The evidence that excessive cognitive demand at work can harm the workers’ health and performance has led to a renewed interest in cognitive load theory (CLT). CLT examines the interaction of cognitive structures, information and its implications (Sweller et al., 1998). Precisely, the term “cognitive load” refers to the amount of processing that performing a particular task imposes on the learner’s cognitive system (Paas et al., 2003). Xie and Salvendy (2000) provide a detailed conceptual framework of human information processing. Definitions of instantaneous load, peak load, accumulated load, average load, and overall load are presented to investigate the trend of cognitive load over time as a response to stimuli that an activity and/or environmental conditions are imposing on the subject. The following sections investigate the current state-of-the-art about mental workload modelling and cognitive cost estimation for performing tasks, according to the three main categories defined at the beginning of Section 2 and displayed in Figure 3.

#### 2.2.1 *Subjective judgements*

Thus far, narrative interviews and subjective rating scales represent the most commonly used method to measure the cognitive load both in laboratory and industrial settings (Rubio et al., 2004; Leppink et al., 2013). NASA-TLX (Hart and Staveland, 1988; Hart, 2006), subjective workload assessment technique (SWAT) (Reid and Nygren, 1988) and subjective workload technique (SWORD) (Vidullch et al., 1991) are just a few of the available questionnaires in the literature. Self-ratings nevertheless have many limitations (Naismith et al., 2015). The main drawback is the assumption that people are able to introspect on the cognitive processes and report the amount of experienced cognitive effort (Naismith et al., 2015). Additionally, they are often affected by many biases, such as acquiescence and social desirability, and their outcome may appear questionable. Lastly, they work offline and the deep comprehension of collected data requires specific skills in the field of cognitive ergonomics and cognitive science.

More recently, researchers presented tools intended to be used directly by workers involved in the manufacturing domain. For instance, the work in Thorvald et al. (2019) presented a factor assessment tool and a handbook, denoted cognitive load assessment for manufacturing (CLAM), to estimate the mental workload that human operators were expected to employ within specific assembly tasks and workstation layouts. End-users were asked to reflect on different aspects of their daily activity and rate factors on a scale from 0 to 8. A specific combination of them resulted in the final cognitive load score.

#### 2.2.2 *Systematic observations*

To assess the cognitive effort in performing industrial activities, two strategies have been adopted based on direct observations of the involved operators: 1) performance measures on either the first or secondary task and the 2) analysis of behavioural characteristics and modifications.

Task- and performance-based techniques involve measurements on both the primary and secondary tasks. The idea behind is that people have limited resources, so tasks performed concurrently are supposed to reflect the level of the cognitive load imposed by the primary task (Kaber and Riley, 1999; Paas et al., 2003; Wu and Li, 2013). State-of-the-art measurements to assess the performance are task time, run time, reaction time, accuracy, and error rate. Despite the high sensitivity and reliability, this technique interferes considerably with the usual task execution, making it rarely applicable, even in laboratory settings.

In an effort to design less obstructive monitoring systems and maximise users’ comfort, the applicability of external sensory systems has been recently examined. Early studies exploited camera sensors for automatic emotion recognition (Glowinski et al., 2011; Karg et al., 2013; Kleinsmith and Bianchi-Berthouze, 2013; Roy et al., 2020) and expression synthesis (Karg et al., 2013; Kleinsmith and Bianchi-Berthouze, 2013), as well as activity-related behavioural indexes of stress (Giakoumis et al., 2012; Aigrain et al., 2015). A quantitative and online framework was developed by Lagomarsino et al. (2022b) to monitor the cognitive workload of human operators by detecting patterns in their motion directly from the input images of a low-cost RGB-D camera. The method examined how industrial work affects people relative to their attention distribution, decision-making, mental overload, frustration, stress and errors. Head pose estimation and skeleton tracking were exploited to investigate the workers’ attention and assess hyperactivity and unforeseen movements. Despite the growing interest in the topic, assessing cognitive load through visual monitoring systems is a moderately new topic (Bisogni et al., 2022) with the potential to bring solutions from the laboratory to the actual shop floor.

#### 2.2.3 *Direct measurements*

A great deal of previous research into cognitive load assessment explored *direct measurements* of physiological signals. Physiological measurement of workload relies on the physical reaction of the human body to an intense mental demand (Sweller et al., 1998). The monitoring of brain activity is the most direct and accepted form to investigate cognitive processes. The electroencephalogram (EEG) provides an online, continuous measure of fine fluctuations in instantaneous mental load (Al-Shargie et al., 2016; So et al., 2017; Yang et al., 2019). Nevertheless, motion artefacts and noise due to electrical interference, breathing and heartbeat make the EEG signal not deployable in industrial settings.

Work-related stress and strain were found to alter sympathetic-parasympathetic nervous system balance and raise the risk of heart diseases (Hughes et al., 2019). Therefore, the short-term cardiovascular consequences of mental work were investigated. In the literature, several heart rate variability (HRV)-derived metrics were defined in the time, frequency and non-linear domain. Typically, cognitive workload leads to a decrease of time-domain measures (e.g. mean RR intervals (Henelius et al., 2009)), as well as a reduction of low frequency (LF, 0.04 − −0.15 Hz) and high frequency (HF, 0.15 − −0.4 Hz) powers, while the ratio LF/HF increases (Durantin et al., 2014; Delliaux et al., 2019).

Besides, the galvanic skin response (GSR, also known as electrodermal activity, EDA) has been widely studied for indexing variations in sympathetic arousal associated with emotion, cognition, and attention (Critchley, 2002; Poh et al., 2010; Setz et al., 2010). GSR or EDA is the measurement of the continuous changes in the skin’s electrical conductance arising from the diverse sweating activity of the human body. Researchers identified two components in high-resolution EDA signal, i.e., the tonic (skin conductance level, SCL) and phasic response (skin conductance response, SCR), and used derived metrics to quantify cognitive states and stressful periods (de Santos Sierra et al., 2011; Kyriakou et al., 2019; Han et al., 2020).

More recent studies also included measures of respiratory activity (e.g., respiratory rate, volume and concentration of CO_2_ in airflow) (Grassmann et al., 2016), eye activity (e.g., eye blink rate, intervals of closure, horizontal eye movement, pupil dilation and eye fixation) (Ahlstrom and Friedman-Berg, 2006; Coyne and Sibley, 2016), cortisol level (Carrasco and de Kar, 2003), and speech measures (e.g., pitch, rate, loudness, jitter and shimmer) (Yin et al., 2008).

Psychophysiological measurements provide objective and quantitative information and permit the visualisation of continuous trends and the identification of detailed patterns of load. Nevertheless, signal acquisition requires expensive and impractical systems that are highly sensitive to human movements and often hinder users’ daily activities. For the above-mentioned reasons, the adoption of the technology in real-world scenarios is subject to certain limitations.

## 3 Human monitoring hardware and systems

**FIGURE 4:**
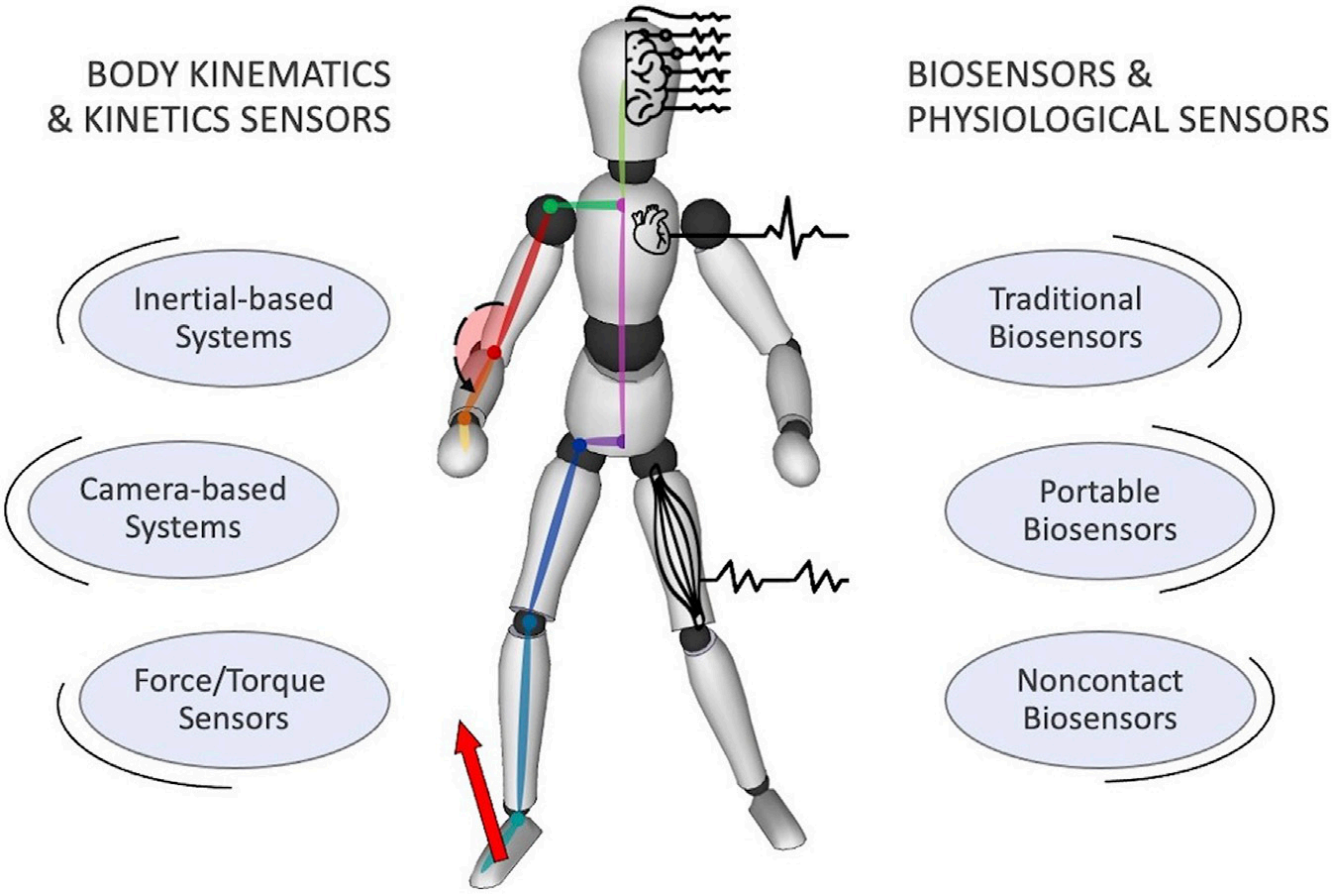
Overview of the different available technologies for the measurement of kinodynamic variables as well as physiological parameters to account for human ergonomics.

In the previous sections, we went through the current standards and methods for ergonomics assessment applicable to the industry. All the presented indexes and algorithms need, to a certain extent, monitoring of human state. We then inspect the literature by searching for human monitoring hardware and systems, by first selecting keywords related to broad concepts (e.g., “human monitoring,” “human sensors,” “biosensors,” “biosignals”) and then specific categories (e.g., “motion-capture,” “electrocardiography,” “electromiography,” “electroencephalography”). Within the scope of this review article, we divide the monitoring systems into two separate groups, based on the measured quantities, namely (i) *body kinodynamics* data and (ii) *biosignals and physiological indicators* (see Figure 4). In each group, the choices of sensors and devices impose challenges that should comply with the rigid rules imposed in industrial settings, namely:

• to guarantee task timing and data synchronization requirements (online measurement);
• to ensure workers’ safety and avoid excessive encumbrance, as well as physical and mental demand (non-invasiveness^4^);
• to ensure the quality of measurement (accuracy and precision).

It can be noted that these indicators have commonalities with those introduced in Figure 2 about the workload assessment. The challenges and the environmental limitations are in fact similar. It is not our interest to address the economical cost comparison of the devices market panorama.

### 3.1 *Body kinodynamics*

Biomechanical analysis requires data on the posture and forces exerted by a specific worker during their duties. It means that online monitoring of body posture and interaction forces is required to estimate the time series data of physical workload.

Human postures are commonly obtained by motion-capture (MoCap) systems. Indeed, realistic skeletal simulation is required to perform the synthesis and analysis of the performed human motions in time. In the synthesis phase, MoCap data leads to improvements in the human model rendering, while the analysis aspects help the researchers in obtaining critical insights into human musculoskeletal systems, such as body joints angles and velocities, movements of body center of mass, body segments poses, etc. Different technologies and solutions have been developed to capture motion, hereafter we analyse some of the most widespread examples in the literature along with some industrial applications. Camera-based systems with infrared (IR) cameras can be used to triangulate the location of retroreflective rigid bodies (markers) attached to the targeted subject (Nagymáté and Kiss, 2018; Chatzitofis et al., 2021; Hu et al., 2021). In addition, systems based on inertial measurement units (IMU) that track the relative movements of articulated structures have become popular for their versatility (Vignais et al., 2013; Caputo et al., 2018; Marín and Marín, 2021). Moreover, at the time of writing, markerless optical MoCap systems undergo significant research progress with high application potential. These systems rely on image processing and deep learning techniques to track human skeletal information online using off-the-shelf and relatively cheap RGB-D cameras (Bortolini et al., 2020; Kim et al., 2021c).

Let us now list some literature examples of MoCap applications in industry. The aforementioned MoCap technologies were exploited, for example in Maurice et al. (2019), where a dataset of human motions in industry-like activities, fully labelled in line with EAWS, is presented. First, this dataset is applicable for classifying, predicting, or evaluating human motions in the industrial environment. Second, it supports the robotics communities to provide collaborative solutions aiming at improving the workers’ ergonomics. In this study, the Xsens MVN Link and Qualisys were used as inertial and marker-based optical MoCap systems. Besides, the data collection procedure was recorded with two video cameras to be further analysed with the OpenPose library (Cao et al., 2021). A similar logistics-dataset was also presented in Niemann et al. (2020) where picking and packing scenarios were recreated to be used in recognition and analysis of human activities in logistics applications.

Currently, the industrial exploitation of these technologies is challenging due to their inherent limitations (Mündermann et al., 2006; Damgrave and Lutters, 2009; Bailey and Bodenheimer, 2012; Lopez-Nava and Angelica, 2016; Patrizi et al., 2016; Colyer et al., 2018; Yahya et al., 2019; Menolotto et al., 2020; Kanko et al., 2021). The need for highly specialised equipment, regular calibration routines, limited capture volumes, inconvenient markers or specialised suits, as well as the significant installation and operation costs of these systems, has so far greatly impeded the adoption of the optical marker-based systems (e.g., OptiTrack™, NaturalPoint, Inc.) in industry despite their compelling accuracy performances (Menolotto et al., 2020). This major drawback can be overcome using the latest advancement in markerless depth-based optical MoCap systems (e.g., RealSense™, Inter Intel Corp.). Accordingly, the users can freely perform their activities and tasks without wearing a suit or having attached markers on their bodies. Nonetheless, up to now, they offer less accurate measurements with respect to the marker-based systems, and they similarly suffer from the occlusion problem. Above all, optical systems in general induce visibility issues due to the limited range of installed cameras. As an alternative, IMU-based systems can track a variety of postures in the cluttered environment associated with both indoor and outdoor applications, indicating higher portability and deployability. In fact, it is not necessary to place or install any fixed infrastructure to use the inertia-based solutions. However, drift, i.e., divergence of the output values from their real values that happens in time, is the common issue with the IMU-based systems that does not ensure an accurate absolute position of the limbs (Damgrave and Lutters, 2009). Besides, artifacts due to skin movement can act as sources of measurement noise during the acquisition of both IMU-based and marker-based systems, which can lead to errors that, in some cases, are of the same order of magnitude as the motions of the studied joints.

On the other hand, to measure the force exerted by a worker, force sensors and force plates are mainly applied (Hoffman et al., 2007; Arjmand et al., 2009). Force sensors can measure the interactions between workers’ hands and the tasks’ objects. In addition, most of the available force plates can simultaneously measure the external ground reaction forces (GRFs) in three planes, i.e. vertical, anterior-posterior, and medial-lateral. However, the installation of the former and the portability of the latter are issues that undermine their industrial adoption. As a solution, wearable alternatives are suggested in the form of gloves (Park et al., 2019) and shoes (Bamberg et al., 2008; Muzaffar and Elfadel, 2020) equipped with force/torque sensors. Instrumented gloves, for example, remove the need to equip handles and tools with force sensors or pressure mats. However, force sensor mats embedded within gloves acquire only normal forces, require calibration and may shift during measurements (Ranavolo et al., 2018). Notably, the wearable insole pressure system can acquire the GRF and plantar pressure data under the foot. Moreover, they can be easily inserted or attached to workers’ safety boots with a minimal hindering level.

### 3.2 *Biosignals and physiological indicators*

As we stated in Section 2.2, investigating physiological signals can be helpful in understanding physiological-related aspects of work, such as mental stress, mental fatigue, and physical stress. The increased interest in these topics aroused from the recent technological progress, making available wireless, off-the-shelf, lightweight, and affordable biosensors.

Biosensors can be classified as 1) *traditional* biosensors; 2) *portable* biosensors; 3) *noncontact* biosensors.

*Traditional* modes of monitoring physiological quantities rely on hospital-based diagnostics devices that, in a controlled environment, guarantee high-quality signals. However, they usually require trained staff and in most cases an offline post-processing phase to extract meaningful features. In this context, more *portable* wearable biosensors emerged with the specific attempt to extend the biosignals’ gathering to nonclinical settings. Recent advancements in technology made available a vast number of biosensors convenient (e.g., headsets, chest straps, and wristbands) that can be deployed to check the ergonomic state of the workers in the field.

Chest-strap sensors were primarily used for monitoring the cardiac activity (e.g., HRV) of workers. Chest straps offer an easy and accurate alternative to traditional electrocardiography (ECG) measurements. Although progress in technology design has reduced the bulkiness of the sensor on the strap, the acceptance and practicality of such a device are still in their initial stages (Hinde et al., 2021). As stated in Section 2.2, a few researchers attempted to assess workers’ mental stress and cognitive load based on brain waves collected from EEG headsets. The main advantage of these wearable devices is that they show compelling rapidity performances in pointing out changes in workers’ mental state fulfilling the online requirements. Nevertheless, capturing high-quality EEG signals in the field is more challenging compared with other physiological signals due to several intrinsic artifacts (e.g., eye blinking and facial muscle movement) (Mijović et al., 2017). Among different wearable biosensors, wristband-type biosensors allow researchers to acquire multiple physiological signals (e.g., photoplethysmography, EDA, and skin temperature) without interrupting workers’ ongoing tasks. However, measuring physiological signals using a wristband-type biosensor at the industrial floor is still challenging because of the large number of extrinsic signal artifacts and distortions that come from workers’ movements, sensor displacement, environmental noises, and the lower quality of sensor electrodes compared with wired biosensors (Jebelli et al., 2019).

Finally, we have *noncontact* sensors that are able to acquire psychophysical signals without any contact between the sensor probe and the human body, guaranteeing the best performances in terms of non-invasiveness. Pupil diameter, gaze data, gaze duration, and eyelid closure patterns can be remotely recorded through infrared eye-gaze tracking systems. Eye-tracking is a functional and highly neuro-ergonomic solution for gathering both mental workload and relevant practical attentional information. It is a non-invasive and easy to set up device, allowing for consistent data collection. Moreover, it does not impose any physical burden on users, and the calibration routine is fast and straightforward. A variety of measures were described in the literature that elucidates the efficiency of visual search related to mental workload, including fixation count, fixation duration, fixation rate, the fixation to saccades ratio, average saccade distance and velocity, peak saccade velocity, number of long fixations, and average pupil diameter (Ahlstrom and Friedman-Berg, 2006). Online processing of the aforementioned metrics does not require significant computational power and is not as complex as EEG or other brain imaging techniques.To recap, with the aim to assess workers ergonomics in industrial environments, the preferred sensor systems should be lightweight, easy to wear and/or set up without hindering workers’ activities and ensuring user comfort even for prolonged usage. Concerning *body kinodynamics*, inertial-based devices provide accurate measurements but may be impractical in some task conditions. On the other hand, external sensor systems, such as cameras, offer non invasive analysis of the human motion but suffer from occlusion and feature limited accuracy. Besides, *biosignals and physiological indicators* provide useful insight about the human state. These have shown a good potential for offline validation of possible ergonomic interventions, but may be not appropriate for online application.

## 4 Ergonomics in human-robot collaboration

**FIGURE 5:**
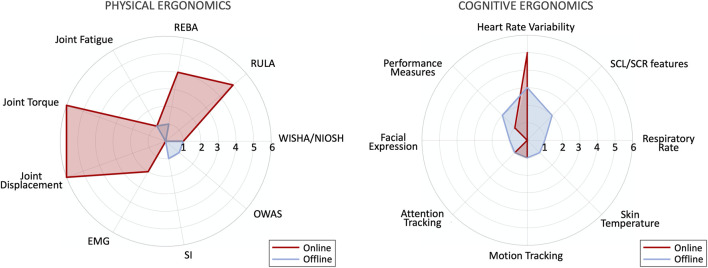
Number of papers related to the main assessment variables in physical and cognitive ergonomics.

This section presents the online compensatory measures and strategies that a robotic partner, fed with the ergonomic evaluation of an operator (Section 2) and/or with the data collected with available monitoring technologies (Section 3), can put in place to mitigate the human workload. To focus the literature analysis on ergonomic collaborative robotics, we set the conceptual boundaries on the terms describing HRC in relation to the industrial sector (e.g., “human-robot,” “collaborative robot,” “manufacturing,” “automation”) and human ergonomics (e.g., “physical,” “cognitive,” “workload,” “stress,” “effort”). The polar plot in Figure 5 illustrates the production of papers linked to our research query over a ten-year time window (from 2011 to 2022).

### 4.1 Physical ergonomics in human-robot collaboration

Within the scope of this review article, we framed the different approaches to address workers’ physical ergonomics during HRC in two macro areas. On the one hand, several authors elected some among the *systematic observations* methods presented in Section 2.1.2 to either identify a more ergonomic human posture or define a cost for task planning and role allocation. Hence, the criteria to drive HRC were selected directly among the gold standard ergonomic tools. On the other hand, assumptions were made by some researchers to indirectly achieve more ergonomic conditions for the workers, e.g. the reduction of human joint torque/fatigue or the optimisation of the human field of view/arm kinematics would improve task ergonomics. For sake of readability, in the following, these two categories are addressed as *standard-based* and *cost-based* approaches.

#### 4.1.1 *Standards-based* approaches

The works that deem ergonomics in HRC in a direct way are presented and grouped in the following according to the observational method employed. Above all, the REBA method was exploited by a bunch of researchers. Busch et al. (2017) first adopted REBA to improve the workers’ comfort and safety during a human-robot collaborative task. The human posture that minimised the score returned by REBA was computed and the robot pose was adjusted online to let the human perform the task in the optimised body configuration. A graphical interface was also developed here to inform the user about the ergonomics evaluation results. The differentiable version of REBA was then introduced in a later work (Busch et al., 2018) to simultaneously design robot motion (in the same way as before) and plan the sequence of actions in the task. The REBA technique was also adopted in Van de Perre et al. (2018) to both predict and optimise human ergonomics during an hybrid co-carrying task. First, the human postures from hands poses were computed for a set of states (i.e., possible configurations to manipulate the object) and an ergonomic cost according to REBA was assigned to each state. Next, the sequence of states was found that minimise this cost, resulting in a joint plan for the two agents that was optimised for human ergonomics. On the other hand, Zanchettin et al. (2019) presented a control strategy to facilitate the human to assume a more convenient body configuration while operating on a bulky object held by the robot. The robot moved the workpiece so that the human was induced to assume his/her most natural and ergonomic posture according to REBA. Finally, El Makrini et al. (2019) employed the REBA to set a criterion for task allocation in a human-robot assembly operation. Each sub-task of the assembly was assigned to either the robot or the human based on the task requirements, the capability of the agents but also the evaluation of the human body posture given by REBA.

Right after the REBA, the most commonly used standard ergonomics tool within HRC is the RULA method, likewise posture-based. Ferraguti et al. (2020) employed RULA within the proposed architecture for human-robot co-manipulation. Different objects were considered during the experiments and positioned in the most comfortable way for the user to operate them, estimated based on the postures with the lower RULA score. In addition, the position of such objects could be conveniently adjusted directly by the user thanks to the robot admittance control. Similarly, Shafti et al. (2019) presented an approach to continuously invoking cooperative robot movements that meet the human partner’s ergonomic postures according to RULA. Conversely, a simulation presented in Zacharias et al. (2011) adopted RULA not to identify ergonomic configurations for the humans but to plan a more human-like robot motion, which appeared safer and more interpretable from the human point of view. Human-robot role allocation is instead addressed by Merlo et al. (2021), which proposed a RULA-based model for physical risk prediction. The developed online strategy can assign actions among the agents (i.e., human or robot) according to a human physical state indicator, called kinematic wear, that can account for the usage of each joint based on RULA guidelines. From the same research group, Fusaro et al. (2021) proposed a method to generate robot plans for both autonomous and human-robot cooperative tasks taking into account human ergonomics. The developed approach allowed the robot to online adapt its plan to the human partner, by choosing the tasks that minimize some execution costs that entailed human availability, decisions, and ergonomics. The latter was specifically addressed by using the WISHA index which, unlike REBA and RULA, considers also the task frequency, duration and the involved weights. Task planning for human-robot collaborative tasks was also addressed in Faber et al. (2016), Liau and Ryu (2020) and Pearce et al. (2018), by determining the ergonomic cost with the OWAS, the RULA and the SI, respectively, but in simulation or offline, at a later stage.

Lastly, it is worth mentioning some studies in which observational methods were employed to evaluate HRC workstations offline (Gualtieri et al., 2020; Colim et al., 2021; Dimitropoulos et al., 2021; Palomba et al., 2021) or in simulation (Castro et al., 2018; Heydaryan et al., 2018; Lietaert et al., 2019; Mateus et al., 2019; Laudante et al., 2020).

#### 4.1.2 *Cost-based* approaches

The studies that indirectly address human ergonomics demands in HRC are illustrated hereafter, grouped according to the quantity employed as a cost. In Bestick et al. (2016, 2015) the most convenient robot configurations when handing over an object to a human partner were investigated. A simple cost function was assumed *a priori* considering the distance of human joints from a neutral position. The higher this ergonomic cost, the less likely the associated human body configuration, leading to a specific robot pose. Instead, in Bestick et al. (2018), the ergonomic cost was learned online via Bayesian inference, based on implicit physical queries from the robot. Katayama and Hasuura (2003) introduced five different optimisation models to characterised human comfort. Among the latter, the “medium joint angle index” was selected by Sisbot and Alami (2012) to model arm comfort, which, along with human safety and visibility, was employed as a criterion to generate ergonomic robot paths toward the object transfer point (OTP) for handover. The extensions of this human-aware manipulation planner took into account also HRC constraints (Mainprice et al., 2011) as well as human mobility (Mainprice et al., 2012).

In these studies, the ergonomic cost selected only consider the kinematics of the human actions. On the other hand, the “joint torque model” proposed in Katayama and Hasuura (2003) was instead employed by Parastegari et al. (2017) to predict an OTP that matches the human receiver preferred position. The overloading joint torque, i.e., the torque induced only by the effect of an external load, were instead employed as a cost in Kim et al. (2018a); Lorenzini et al. (2018); Kim et al. (2019, 2021b) to optimise the human body configuration in co-carrying or co-manipulation activities according to human stability, shared workspace and task constraints. The cumulative effect of the overloading joint torque, i.e., overloading fatigue, was then considered in a later work (Lorenzini et al., 2019) to trigger the adaptive robot behaviour at the onset of physical fatigue.With the aim to plan a shared human-robot assembly task, in Michalos et al. (2018) a multi-criteria method was proposed that employs a set of quality, productivity but also ergonomics criteria to identify the best planning scenario in a simulated environment. A fatigue model as well as the NIOSH index were exploited here as costs for the search algorithm. Lamon et al. (2019) introduced a set of indexes to evaluate agents performance combined with an offline allocation algorithm for optimal role allocation in an industrial assembly task. Among these indexes, agent dexterity and effort were proposed to consider human comfort and physical fatigue thus ergonomics from both a kinematic and dynamic point of view, respectively. In the last two studies, the fatigue model was based on the one proposed in Ma et al. (2009).

The multiple ergonomic indicators defined by Maurice et al. (2017) were employed to orient the design of a CoBot, but the proposed set of variables, due to their ease of computation, may be employed online for HRC. Similarly, in Rapetti et al. (2019) an optimisation problem was formulated, where ergonomics targets such as muscular effort and body posture were mapped to human kinodynamic quantities such as joint torque and joint angles/velocities, to develop a human-aware robot controller for HRC.

Alternatively, in Marin et al. (2018) the optimisation of “contextual ergonomics models” was proposed to successfully reduce the muscle activation of subjects performing a drilling task. The presented models were Gaussian process latent variable models trained offline with detailed musculoskeletal simulations but can be employed in a low-dimensional latent space, featuring potentially online capabilities.

Lastly, human effort can be expressed through physiological measures. In this view, Peternel et al. (2017, 2018) presented a method to adapt online the robot behaviour to human fatigue, which was modelled based on human muscle activity measured with EMG sensors.

### 4.2 Cognitive ergonomics in human-robot collaboration

In addition to ensuring the operator’s physical safety and comfort, the cognitive resources demand and mental stress induced by the close interaction with a CoBot should not be overlooked. Early interviews with the potential future users (i.e., actual industrial workers) of these robots revealed controversial attitudes and social cues (Wurhofer et al., 2015; Elprama et al., 2016, 2017; El Makrini et al., 2018). This underlies the need to gather quantitative data giving insights about the mental processing system.

A number of cross-sectional studies recorded the physiological activity of the human during industrial-mimicking tasks in laboratory settings and post-processed the acquired biosignals to appraise how cognitive load develops during HRC. Novak et al. (2011) assessed physical and cognitive load during haptic interaction with a robot by measuring ECG, GSR, respiratory rate and peripheral skin temperature. The combination of the last two measurements permitted estimating the mental cost in physically demanding tasks with haptic robots. Nevertheless, the proposed setup requires impractical equipment (i.e., a thermistor flow sensor), making the technique hardly deployable in the industrial sector. Arai et al. (2010) measured the mental strain in human-robot collaborative assembly tasks by recording the skin conductance and asking participants to fill a subjective questionnaire. An increase in psychophysiological conditions was found when a robot moved closer to a worker, with high approaching speed and without notifying its motion in advance. Analogous results were achieved by Kulić and Croft (2005, 2007a,b) exploiting a more comprehensive range of physiological measurements (i.e., heart rate, corrugator muscle activity, GSR) and by Höcherl et al. (2017) and Bergman and van Zandbeek (2019) through *subjective judgements*.

The interrelations between cognitive fatigue, operator sex and robot assistance level were also examined to optimise HRC system designs with respect to task performance and user experience. According to Hopko et al. (2021), the cognitive effort, measured by HRV signals, had a negative impact on task efficiency but did not change accuracy or precision. Instead, the assistance through automation was subjectively perceived and rated in questionnaires as benefitting performance in female subjects. Results of *subjective judgements* and secondary-task performance in Kaber and Endsley (2004) indicated that, when a more significant percentage of primary task time was automated, operator perceptual resources were freed-up and monitoring performance on the secondary task improved. In addition, Héraïz-Bekkis et al. (2020), Dragan et al. (2013), and Höcherl et al. (2017) found that the feeling of perceived safety was enhanced when the robot motion was fluent and predictable. More recent attention has focused on the provision of smooth trajectories to generate psychologically acceptable motions without adding disturbances or uncomfortable sensations to the worker (Rojas et al., 2019, 2021). However, variations in perceived level of cognitive workload with the proposed robot’s control strategies has not been tested yet.

Lately, three machine learning modalities were presented and compared in Rajavenkatanarayanan et al. (2020) to extract relevant features from ECG and GSR data, collected during robotic-assisted assembly tasks. The support vector machine (SVM) provided the best accuracy of 92.85% in classifying the cognitive ergonomic risk (i.e., low or high) potentially online.

With the aim to not introduce an additional source of discomfort for the worker (e.g., wearability constraints introduced by biosensors) in manufacturing activities, Lagomarsino et al. (2022a) monitored the mental effort and stress level of human operators by detecting patterns in their motion directly from the input images of a stereo camera. In particular, the online and quantitative framework examined operators’ attention distribution, high activity periods and body language while interacting with an industrial CoBot. The degree of robot’s transparency and observability provided to the human worker had an influence on the development of cognitive workload. Moreover, changes in experienced mental effort between human and automated assistance were mainly correlated to the operator’s familiarity with the technology (Wurhofer et al., 2015). The transparency of robot behaviour was also considered by Dragan et al. (2013), Faber et al. (2016) and Gombolay et al. (2017) as criteria influencing cognitive ergonomics and included in the algorithm for ergonomic role allocation in hybrid industrial settings. In addition, Eimontaite et al. (2019) explored the impact of graphical signage on participants physically collaborating with a semi-autonomous robot. According to data collected by questionnaires and facial expression recognition, participants reported decreased anxiety levels and negative attitudes toward the robot. On the other hand, the task-relevant signage supported task performance accuracy rate and response time.

All of the studies reviewed here aim to appraise the impact of industrial CoBots and their actions on operators’ mental states in hybrid environments. The works of Lambrechts et al. (2021) and Gualtieri et al. (2021a) identified multiple cognitive ergonomics variables in human-robot collaborative systems and underlined the importance of monitoring the human state and interpreting nonverbal communication. Indeed, this information can be exploited to design frameworks capable of enhancing the interaction between humans and robots by adapting online the behaviour of the robotic teammates to operators’ needs. The HRC solutions would build the foundations for improving cognitive ergonomics at work and mitigating the burden of work-related mental disorders worldwide.

Research following this principle is usually referred to as “affective robotics” (Braezeal et al., 2016), though current approaches are mainly devoted to social or service robots. Moreover, the researchers are encountering difficulties due to the multidimensional construct and high subjectivity of cognitive processing and the typical human attitude of being ashamed and concealing about psychological state. In Nicora et al. (2021), a human-driven control architecture including a CoBot and an interactive virtual Avatar is envisioned for promoting good mental health. Preliminary attempts of introducing affective robotics in industrial settings were presented by Landi et al. (2018); Villani et al. (2018b, 2020). The proposed system estimated the user’s mental fatigue by analysing HRV and online tuned the velocity of the slave, forbade hazardous manoeuvres or provided assistive forces at the master interface. The interesting study by Messeri et al. (2021) offered a novel HRC paradigm where the CoBot adapted its behaviour online based on the mutual evaluation of the operator’s stress and productivity. More specifically, the actual HRV parameters and cycle time were compared to the reference values retrieved by a game theory approach. The outcome was then exploited at each task execution pipeline loop to vary the pace of interaction for simultaneously mitigating the human cognitive workload and maximising productivity. The trade-off between system productivity and acceptable amount of human cognitive workload in industrial tasks was also tackled by Lagomarsino et al. (2022c). A multi-objective optimisation problem and an online HRV-based decision-making algorithm were implemented to tune the total execution time and smoothness of the trajectories accomplished by the CoBot. This permits finding the most appropriate pace of interaction for each specific user and online adapting CoBot’s motion characteristics to fulfil changes in the individual needs.

Despite the growing concern for employees and employers worldwide in work-related stress and psychosocial risks, our careful investigation identified Lagomarsino et al. (2022a,c); Landi et al. (2018); Messeri et al. (2021); Nicora et al. (2021); Villani et al. (2018b, 2020) as the only studies which attempt to tackle the challenge of online assessing the cognitive demands and mitigating pressures at work.

## 5 Discussions and outlook

The review shown in the previous section provides evidence of the emerging and attractive interest in integrating ergonomic appraisals, from both the physical and cognitive/organisational points of view, with robotic strategies to mitigate the estimated hazards. This section first presents the main scientific contribution of this paper, i.e., the identification of the main gaps and thus potential research topics in the integration of ergonomics principles within HRC frameworks. Next, the operational implications are discussed and the practical contribution is highlighted. Then, some insights for possible future studies are provided and last, the limitations of this study are illustrated.

### 5.1 Identification of the gaps

Tables 1, 2 report the allocation of the leading research themes according to the monitoring systems and the ergonomic assessment method in the last 10 years (from 2011 to 2022). As can be seen from Table 1, posture-based observational methods (such as RULA and REBA) are already widely studied to assess physical ergonomics in HRC and therefore known and consolidated from a research point of view. Nevertheless, as already mentioned, such techniques are limited to the kinematics of the workers’ actions. Just a couple of researchers considered NIOSH/WISHA and SI, which take into account also the task frequency, duration and the weight of the involved objects. On the other hand, pen-and-paper comprehensive techniques (i.e., including loads, action forces, repetitions, etc., such as EAWS) have been already proposed in an automatised version but have not found their practical application so far as a policy for HRC. In general, however, the existing standard ergonomic tools present the great limitation of not plenty considering the dynamics of the workers’activities, as discussed in Section 3. The authors who combined *direct measurements* from motion and force sensors with human models always proposed relatively simple metrics to address subjects’ ergonomic demands (e.g., comfortable body configurations or decreased joint torque/fatigue). Indeed, reduced-complexity human models with a limited number of parameters allow fast identification processes and ensure minor computational costs.

**TABLE 1:** Physical ergonomics in industrial HRC: distribution of works among main monitoring systems and ergonomic assessment techniques.

Physical ergonomics in human-robot collaboration		*Systematic observations*		*Direct measurements*	
		Pen-and-paper		Model-based	
		Posture-based	Comprehensive	Motion and forces	Electromyography (EMG)
Kinodynamics	Inertial-based Systems	Shafti et al. (2019), Van der Spaa et al. (2020), Merlo et al. (2021)		Kim et al. (2018a), Kim et al. (2021b), Kim et al. (2019), Lorenzini et al. (2018), Lorenzini et al. (2019), Rapetti et al. (2019)	
	Camera-based systems	Bestick et al. (2015), Busch et al. (2017), Busch et al. (2018), Pearce et al. (2018), Zanchettin et al. (2019), El Makrini et al. (2019), Ferraguti et al. (2020), Fusaro et al. (2021)		Mainprice et al. (2011), Mainprice et al. (2012), Sisbot and Alami. (2012), Marin et al. (2018), Parastegari et al. (2017)	
	Force sensors			Kim et al. (2018a), Kim et al. (2021b), Kim et al. (2019), Lorenzini et al. (2018), Lorenzini et al. (2019)	
Physiological	Traditional Biosensors				Peternel et al. (2017), Peternel et al. (2018), Peternel et al. (2019)
	Portable Biosensors				
	Noncontact Biosensors				

**TABLE 2:** Cognitive ergonomics in industrial HRC: distribution of works among main monitoring systems and ergonomic assessment techniques.

Cognitive ergonomics in human-robot collaboration		*Systematic observations*		*Direct measurements*	
		Performance measures	Behavioural assessment	Physiological measures	
				Electrocardiography (ECG)	Galvanic skin response (GSR)
Kinodynamics	Inertial-based systems				
	Camera-based systems		Eimontaite et al. (2019), Héraïz-Bekkis et al. (2020), Lagomarsino et al. (2022a)		
	Force sensors				
Physiological	Traditional Biosensors	Messeri et al. (2021)		Novak et al. (2011), Rajavenkatanarayanan et al. (2020), Hopko et al. (2021), Messeri et al. (2021)	Novak et al. (2011), Rajavenkatanarayanan et al. (2020)
	Portable Biosensors			Lagomarsino et al. (2022c), Landi et al. (2018), Villani et al. (2018b), Villani et al. (2020)	
	Noncontact Biosensors				

By offering online monitoring of both human kinematics and dynamics, this approach allows an exhaustive and off-the-shelf evaluation of the worker physical workload, while meeting the requirements of the real factories. A few works exploit muscle activity measured by EMG sensors to model physical fatigue in hybrid environments. Despite their application limits in industrial settings, biosensors and bodily signals could provide worthwhile information to enable the monitoring of the human-robot pair.

Table 2 demonstrates that physiological indicators are instead broadly explored for assessing cognitive workload, even if no well-established methodologies or commonly accepted metrics for the cognitive workload exist in the literature. Review results reveal a growing interest in unobtrusive sensing and wearable devices. The latter permits the (i) simultaneous monitoring of multiple physical and physiological signals, sensitive to distinctive aspects of workload, and their (ii) fusion to obtain more reliable insights into human fatigue and cognitive processes.

Behavioural analysis is generally less investigated but, according to the growth of paper production in recent years, it represents an emerging research field in the context of automatic detection of stress, frustration and anxiety. This is motivated by the advantages of low cost and operational ease of the assessment techniques. However, some disadvantages such as vulnerability to motion and lack of burden-free calibration solutions have not been completely addressed yet. In addition, due to their intrinsic properties, the authors identify noncontact sensors and cameras, maturing awareness and protection of privacy, as potential sensing systems that scientists should concentrate and focus on to develop the next generation of industrial CoBots taking into account workers’ ergonomics.

### 5.2 Practical implications

Crucial to the integration of what we would call “ergonomic HRC frameworks” in real factories is the applicability and acceptability of the proposed solutions. The applicability is related to the compatibility of the employed sensor systems within the industrial environment, which are often noisy, cluttered, and subject to frequent modifications. As discussed in Section 3, to monitor the human state, both wearable and external devices are available, which can be preferable depending on the tasks to be executed and the work place characteristics and requirements. In general, the sensor technologies should be selected to ensure a reliable monitoring of the workers’ state but also maximise their comfort without hindering daily activity. Indeed, the acceptability is related to workers’ opinion. Since they will firsthand use and experience the proposed technologies, their approval is fundamental for an effective integration. Hence, parallel to the development of HRC strategies, their evaluation with real workers in real factories is a key requirement. To date, several questionnaires are available for this purpose, e.g., the system usability scale (SUS) (Brooke, 1996), Borg scale (Borg et al., 1985), Positive and Negative Affect Schedule (PANAS) (Thompson, 2007) and also the NASA-TLX (Hart and Staveland, 1988; Hart, 2006), but should be updated to keep apace with the technological advancements. Even the most efficient and ergonomic equipment, without users’ approval, become meaningless. We believe that this review paper can be useful not only for researchers willing to discover new research themes but also to business executives and employee representatives to get an overview about the state-of-the-art of HRC solutions to address human factors and ergonomics. A continuous dialogue of robotics (but non only) researchers with these entities to gather information about applicability and acceptability as well as make them aware of the available possibilities in terms of technology is fundamental for advancements in this field.

On the other hand, to work along the right lines, robotics researchers should receive counselling and support from ergonomics specialist and practitioners to ensure the correct use and applications of ergonomics principles. The exchange of information between these two domains is currently too scarse while it would enhance and fasten the integration of human ergonomics within HRC. By providing simultaneously an overview of the current tools to assess human ergonomics along with the available technologies to monitor the human state, up to their combination in the design of HRC frameworks, we hope to highlight the importance of the constant interplay between the ergonomics and robotics communities.

### 5.3 Future research trends

Despite the growing enthusiasm to understand the development of MSDs and the multidimensional construct of the mental workload, the study of human ergonomics in collaborative robotic workstations is relatively a new topic, still looking for practical solutions. Most of the studies on focus on the impact of industrial CoBots and their actions on operators’ physical and mental states. The literature collection and analysis presented in this article shows that the actual research tends to rely on elaborate and time-consuming data post-processing. Consequently, available tools can be used almost exclusively by experts or merely provide subjective and offline insights about human ergonomics, inhibiting their applicability in real-world environments. Preliminary attempts to gather online data and accordingly adapt robot behaviour are investigated. Although limited to laboratory experiments, results show excellent potentials in mitigating work-related biomechanical and cognitive workload without introducing new occupational safety and health hazards. From a physical point of view, the existing literature offers a variety of ergonomic metrics that became well-established tools in the industrial environment. In parallel, advanced modelling and estimation algorithms can make human kinodynamic variables available online, but most of the underlying techniques are still confined in laboratory settings. As such, the most significant and prominent research themes for physical ergonomics are the integration and transfer of these methods in the workplace. To this end, the potential of wearable sensor technologies should be exploited to maximise users’ comfort without hindering workers’ daily activity. Moreover, future research should proceed with implementing role allocation strategies in hybrid environments and online planning and adaptation of robot movements. All those solutions fall within the cutting-edge principle of designing human-centred workstations supported by automation and could build the foundation for a more inclusive industrial environment. Thanks to the introduction of ergonomics assessment in the control loop, the robot behaviour could be adapted to workers’ physical condition and characteristics (e.g., age, gender, dominant and vulnerable limb, disabilities, fatigue) and enhance specific skills, mitigate risks, fulfil required capabilities and fight inequalities.

To date, research on ergonomics in the industrial sector mainly aims at mitigating the worker’s fatigue and discomfort from a physical point of view. Future research should entail cognitive ergonomics variables, whose implications are still too often undervalued (HSE, 2020). Industrial collaborative technologies provide unique opportunities, but they may perilously increase operators’ mental demand when inadequately handled and result in adverse health and safety hazards. Assuring the acceptability of robotic systems from human workers and guaranteeing perceived safety are the first steps for a successful workplaces’ digitalisation. However, the scientific and industrial communities still need to be provided with a well-structured and validated set of models and metrics for the cognitive workload. Then, in the near future, researchers should concentrate on developing reliable methodologies of the mental demand inflicted by industrial tasks. This could be exploited afterwards in new research lines aimed to maximise workers’ efficiency and workstation productivity and facilitate the adoption of CoBots in real-world industrial environments.

### 5.4 Limitations of this study

The review covered four out of five sub-parts identified by Hendrick (1998); Hendrick and Kleiner (2002) within the field of human factors/ergonomics, i.e., human-machine interface, human-environment interface, human-software interface, human-job interface technologies. These technologies, which are primarily focused at the individual level, are often referred to in the literature as “microergonomics.” The exclusion of the structural dimensions of work systems, i.e., human-organisation interface technology or “macroergonomics,” was mainly motivated by the desire to investigate ergonomic metrics that could be exploited to drive and adapt the robot behaviour and potentially improve ergonomics in hybrid environments. The boundaries of our analysis permit to comprehensively investigate and discuss existing ergonomics assessment tools and available monitoring devices. Nonetheless, systematic macroergonomic methodologies provide a larger perspective of the overall work system and could increase the likelihood of the microergonomic interventions presented in this review having a more significant impact and effectiveness.

## 6 Conclusion

The goal of this article was to provide an overview of the current state-of-the-art in ergonomic HRC in industrial settings. Ergonomic assessment methodologies and available monitoring technologies to online adapt robot control strategy according to workers’ distress and needs were investigated, and the most promising research themes were highlighted. Despite the booming attention in physical and cognitive ergonomic HRC, several challenges are still waiting to be solved. In particular, when the technologies for ergonomics monitoring and HRC will reach a more mature level, the challenges to be addressed include the cost-effectiveness, the level of expertise needed to implement and maintain them, and the multi-person examination capacity. These challenges are not only limited to the technical aspects but also to the regulatory ones, such as privacy issues when it comes to monitoring of humans.

## Footnotes

1 With the term “online” we refer to methods and procedures that respond in a timely manner, but with no particular limit on time latency (which are instead required for “real-time” systems and can be ensured only following specific technological requirements).

2 These methods are called “pen-and-paper” since they were originally conceived as hand-written means, but, at present, their digital version is available.

3 The term “kinodynamic” measurements is used, in this article, to indicate the variables associated with both human kinematics (i.e., positions, velocities, and accelerations) and dynamics (i.e., quantities related to interaction forces).

4 With the term “non-invasiveness” is intended the absence of physical constraints or interference to the subject.
